# Reducing posttraumatic stress in parents of patients with a rare inherited metabolic disorder using eye movement desensitization and reprocessing therapy: a case study

**DOI:** 10.1186/s13023-021-01768-7

**Published:** 2021-03-10

**Authors:** Thirsa Conijn, Lotte Haverman, Frits A. Wijburg, Carlijn De Roos

**Affiliations:** 1 Pediatric Metabolic Diseases, Emma Children’s Hospital and Amsterdam Lysosome Centre “Sphinx”, Amsterdam UMC, University of Amsterdam, H8-264, Meibergdreef 9, Amsterdam, The Netherlands; 2 Psychosocial Department, Emma Children’s Hospital, Amsterdam UMC, University of Amsterdam, Amsterdam, The Netherlands; 3 Department of Child and Adolescent Psychiatry, Amsterdam UMC, University of Amsterdam, Amsterdam, The Netherlands

**Keywords:** PTSD, Trauma, Parents, EMDR, Inborn errors of metabolism

## Abstract

Parents of children with severe inborn errors of metabolism frequently face stressful events related to the disease of their child and are consequently at high risk for developing parental posttraumatic stress disorder (PTSD). Assessment and subsequent treatment of PTSD in these parents is however not common in clinical practice. PTSD can be effectively treated by Eye Movement Desensitization and Reprocessing (EMDR), however no studies have been conducted yet regarding the effect of EMDR for *parental* PTSD. EMDR is generally offered in multiple weekly sessions which may preclude participation of parents as they are generally overburdened by the ongoing and often intensive care for their child. Therefore, we offered time-limited EMDR with a maximum of four sessions over two subsequent days to two parents of mucopolysaccharidosis type III (MPS III) patients to explore its potential effects. Both qualitative and quantitative outcomes were used to evaluate treatment effects. Both parents felt more resilient and competent to face future difficulties related to the disease of their child, and no adverse effects were reported. Quantitative outcomes showed a clinically significant decrease in post traumatic stress symptoms and comorbid psychological distress from pre- to post treatment, and these beneficial effects were maintained at follow-up. In conclusion, time-limited EMDR may be a highly relevant treatment for traumatized parents of children with MPS III, and probably also for parents of children with other rare progressive disorders. Further research is needed to validate the efficacy of EMDR in this specific population.

## Background

Inborn errors of metabolism (IEMs) constitute the largest group of disorders in childhood causing progressive intellectual and neurologic deterioration, often in the absence of a disease modifying treatment [1]. Although these disorders are individually rare, their combined prevalence is substantial [2]. Earlier studies showed that IEMs have a highly negative impact on the psychosocial functioning of parents [3–6], and that parents of children with IEMs report a lower health related quality of life compared to parents of other chronically ill children, including pediatric cancer [7]. Parents frequently face potential traumatic medical events (e.g. during the diagnostic phase, but also related to palliative treatment and procedures related to clinical trials) followed by short- or long term stress responses [8]. Consequently, these parents are pre-eminently at risk for developing parental posttraumatic stress disorder (PTSD) [9–11]. According to the 5th edition of the Diagnostic and Statistical Manual of Mental Disorders (DSM-5), facing a life-threatening disease in one’s child indeed qualifies as an event that may lead to PTSD [12]. Parental PTSD is diagnosed when parents fulfil PTSD criteria related to their child’s illness and experience symptoms such as intrusions, avoidance, negative alterations in mood and cognitions, and hyper arousal [9, 12].

Although the assessment of parental PTSD related to severe pediatric diseases such as cancer has gained significant interest [10, 13, 14], studies focusing on PTSD in parents of children with rarer diseases such as IEMs are lacking. Evaluating the presence of PTSD may less often be considered when parents are continuously exposed to stressful events, in contrast to parents of pediatric patients who have experienced more delineated traumatic events (e.g. a restricted period of intense treatment or an acute hospital admission). Therefore, we recently assessed posttraumatic stress symptoms in parents of mucopolysaccharidosis type III (MPS III, Sanfilippo syndrome) patients [15], a rare lysosomal storage disorder characterized by developmental delay from the age of 2–4 years often with severe behavioral problems and subsequent progressive mental deterioration leading to severe dementia and premature death [16]. We found a remarkably high prevalence of PTSD (22%, compared to 3.8% in the general Dutch population [17]) in parents of MPS III patients, underpinning the need for effective trauma treatment [15].

Until now, over 30 randomized controlled trials (RCT) have demonstrated the efficacy of Eye Movement and Desensitization and Reprocessing (EMDR) in reducing symptoms of posttraumatic stress [18]. Studies on the clinical utility of EMDR for *parental* PTSD have, however, not yet been conducted. This is remarkable, as parental PTSD also has a significant influence on the psychosocial wellbeing of the child [19], and may be characterized by other treatment effects. In addition, EMDR is generally provided in weekly sessions which may preclude participation of parents of MPS III patients, as they are often overburdened by the complexities of parenting their child [4, 20]. In order to make treatment more accessible, we offered four sessions of EMDR (1.5 h each) scheduled on two subsequent days, to two unrelated parents of MPS III patients. We designed this case study to explore the value of time-limited EMDR therapy for traumatized parents of progressively ill children, thereby obtaining data that can be used for future studies.

## Methods

### Participants and procedure

The two participating parents (a mother and a father from different families) were recruited by the Dutch expertise center for MPS III (Amsterdam University Medical Centers). The age of the children was approximately 10 years. The children were diagnosed when they were 3 and 4 years old. Parents were referred to the psychology unit by their metabolic pediatrician for treatment of presumed posttraumatic stress symptoms and had not previously received trauma treatment or other formal psychological therapy. The parents visited the hospital on three separate occasions; once for the intake session (1.5 h) and twice for EMDR (2 times 1.5 h per day). EMDR was provided by two licensed clinical psychologists (LH and CR), who have completed accredited training in EMDR (LH: level II, CR: licensed EMDR Europe Child and Adolescent trainer). Written informed consent from parents to describe their cases in the literature was obtained. Minor details have been amended to ensure patient confidentially.

### EMDR therapy

EMDR therapy was delivered following the standard eight-phase protocol [21, 22]. During the intake session, history taking consisted of a standardized case conceptualization assessing a hierarchy of stressful memories and flash forwards (a mental representation of a feared catastrophe) related to the disease of their child. During EMDR, parents were asked to focus on the most distressing image of the selected memory (target image) or flash forward, eliciting the dysfunctional negative cognition (NC) related to the target image, as well as accompanying emotions and somatic sensations. In the desensitization phase, parents focused on the target memory, while simultaneously focusing on an external distracting visual or tactile stimulus. At regular intervals the parents had to rate the target memory with the subjective units of disturbance (SUD) score, with 0 = ‘no disturbance’ and 10 = ‘worst disturbance possible’, until the target memory was no longer disturbing (SUD = 0) and a functional cognition was rated a 7 on the 7 point Validity of Cognition (VOC) scale, with 1 = ‘totally unbelievable’ and 7 = ‘totally believable’. At the end of the session the therapist checked for and processed any residual disturbing body sensations, followed by a positive closure and evaluation [21, 22].

### Assessments

Parents completed reliable, validated questionnaires to measure posttraumatic stress symptoms (related to the disease of their child) and comorbid psychological distress prior the start of EMDR (T0), 1-month post treatment (T1) and at follow-up (T2, mean duration of 6 months after treatment).

Severity of posttraumatic stress symptoms was measured by the Dutch Impact of Event Scale-Revised (IES-R; Kleber & De Jong, 1998) [23, 24]. The IES-R consists of 22 items, rated on a four-point scale according to how often each posttraumatic stress symptom has occurred in the past 7 days (0 = not at all, 1 = rarely, 3 = sometimes, 5 = often). The total score ranges from 0 to 110, where a higher score indicates more posttraumatic stress symptoms.

Comorbid psychological distress was measured using the Brief Symptom Inventory (BSI) [25, 26]. The BSI consists of 53 items that assesses different psychological symptoms (including the symptom dimensions Somatization, Obsession-Compulsion, Interpersonal Sensitivity, Depression, Cognitive issues, Anxiety, Hostility, Phobic anxiety, Paranoid ideation and Psychoticism) rated on a five-point Likert scale (0 = none, 1 = some, 2 = quite, 3 = quite a lot, 4 = extremely). The total score consists of the mean score on all items, where a higher score indicates more symptoms.

### Statistical analysis

First, the qualitative data of the case conceptualization, content of the EMDR sessions and effects reported by parents were described. Second, the reliable change index (RCI), which controls for coincidence or error, was calculated for pre- to post treatment change scores on the IES-R and BSI. RCIs > 1.96 indicates a significant change at *p* < 0.05, suggesting a reliable change. In the current study, a *clinically* significant change is considered when the post treatment score falls within the range of the mean score minus/plus two standard deviations of the normative population [27]. To calculate the RCI for posttraumatic stress scores (IES-R), the SD and the test–retest reliability (α) of the norm scores of Olde et al. [28] were used, with SD = 13.0 and α = 0.88. To calculate the RCI for comorbid psychological distress (BSI), the SD and the test–retest reliability (α) of the norm scores of De Beurs et al. [29] were used, with SD = 0.72 and α = 0.97.

## Results

### Qualitative results: case conceptualization and EMDR sessions

#### Case 1 (father)

The most important symptoms reported by the father were irritability, inability to tolerate bright light and loud sounds, sleeping problems, fatigue, troubles concentrating and remembering, sadness, and feelings of guilt towards a healthy sibling. The disturbing memories and flash forwards reported by father are listed in Table 1. During the first day of EMDR therapy (two sessions), three out of four traumatic memories and flash forwards were processed. The residual flash forward was processed at the first session of the second treatment day. The fourth session was superfluous, due to the fact that all memories and representations had been neutralized. The total duration of the EMDR therapy was 4.5 h. The father reported feeling very surprised by the positive effect of the treatment in such a short period of time. He felt less easily irritated, tolerated bright light and loud sounds better and was more able to concentrate at work. He reported to feel better equipped to balance the care between his children. He divided the attention for the ill child and healthy sibling more equally with his partner, which made him feel less guilty. Fatigue was still present, but he did not experience feelings of sadness anymore. He expressed to feel resilient and competent to face future difficulties related to the disease of his child. No adverse events occurred.

**Table 1:** Most stressful memories and flash forwards related to the IEM of their child

Case 1 (father)	Case 2 (mother)
*Stressful memories*	
Comforted the child in the hospital, saying that everything would be okay after a minor ENT operation (grommets). Now the diagnosis MPS III is known, it became clear that ‘everything would not be okay at all’ (failure as parent, SUD 7)	The pediatrician communicated the diagnosis MPS III to the parents (SUD 9)
The pediatrician communicated the diagnosis MPS III to the parents (SUD 6)	Termination of a subsequent pregnancy because the fetus was diagnosed with MPS III (SUD 8)
	Announcement from the hospital that the clinical trial (enzyme replacement therapy [30]), in which the child participated, was prematurely terminated (loss of hope, SUD 7)
	Attending the funeral of another MPS III patient (SUD 6)
*Flash forwards*	
Funeral of their child with MPS III (SUD 9)	Her child in a vegetative state with palliative care by deep sedation and withholding of fluids (SUD 10)
Image of the child in a special disability-inclusive transport necessitating a lot of medical equipment (tubes) and making repetitive movements and screaming sounds (SUD 8)	Her child in a wheelchair, no longer able to communicate by laughing, eye contact or movements (SUD 8)
	Sudden death of the child (SUD 7)

*SUD* subjective units of disturbance score

#### Case 2 (mother)

At baseline, this mother reported the following psychological symptoms: irritability, difficulties with concentrating and completing tasks, binge eating, sadness, worrying, and not being able to enjoy the interaction with her children. The disturbing memories and flash forwards she reported during the intake session are presented in Table 1. All disturbing memories (four) were fully processed during the first treatment day (highest SUD score first). The two flash forwards were processed during the second treatment day. She reported an immediate effect after the first treatment day. She felt more cheerful, was less easily irritated, was able to stop binge eating and managed to finish tasks. Moreover, she could now also focus on the healthy sibling and was able to enjoy the interaction with her children. She still suffered from fatigue, but did not needed to sleep during the day anymore. Finally, she now could ignore worrying thoughts and felt more competent to handle future stressful events related to the illness of her child. No adverse events occurred.

### Quantitative results

#### Case 1 (father)

Measurement post treatment (T1) showed a clinically significant decrease in posttraumatic stress symptoms and comorbid psychological distress compared to T0 (Table 2). Improvement was maintained at 3 months follow up (T2).

**Table 2:** Reliable change index (RCI) for posttraumatic stress and comorbid psychological distress

	T0	T1	T2	RCI		
IES-R total score (0–110)				T0–T1	T0–T2	T1–T2
Parent 1	67	24	12	6.75*	8.64*	1.88
Parent 2	62	12	3	10.36*	10.52*	.16
BSI total mean score (0–4)						
Parent 1	1.40	.30	.40	6.24*	5.67*	.57
Parent 2	1.53	.28	.26	7.09*	7.02*	.11

^*^Significant decrease between measurements at *p* < .01

#### Case 2 (mother)

Measurement post treatment (T1) showed a clinically significant decrease in posttraumatic stress symptoms and comorbid psychological distress compared to T0 (Table 2). Improvement was maintained at 9 months follow up (T2).

## Discussion

This study reports the effects of a time-limited EMDR therapy in two parents of unrelated MPS III patients. A maximum of four sessions of EMDR scheduled over two subsequent days resulted in a significant decrease of posttraumatic stress symptoms and comorbid psychological distress in both. Moreover, no adverse effects were reported.

The size and persistence of the effects at follow up in our study are remarkable, especially in the context of the progressive and grim course of MPS III, generally causing ongoing daily stress for the whole family [4, 15]. Most PTSD patients who are treated with EMDR are trying to cope with traumatic symptoms resulting from past events, delineated in time, whereas parents of progressively ill children will experience multiple traumas during the course of the disease, and may even more suffer from threats of expected future medical crises and an early death.

The traumatic memories and flash forwards outlined in this study may provide valuable information for medical and psychological professionals about potential traumatic events for parents of children with rare progressive disorders. Since both parents indicated that receiving the diagnosis of their child was still one of the most stressful memories, we are convinced that direct referral to a health psychologist at the time of diagnosis should be standard care, which is common practice in less rare childhood disorders. Our data also reveals the importance of identifying flash forward representations related to future ‘worst-case scenarios’, which was also recently highlighted in a pilot study on the effect of EMDR in multiple sclerosis patients [31]. Identifying and treating flash forward representations may be essential for a successful EMDR treatment for parents of progressively ill children.

In general, our findings in combination with the high prevalence of parental PTSD in this population underline the importance of structural screening for posttraumatic stress symptoms, for example by using Patient Reported Outcome Measures (PROMs). Early and effective treatment of parental PTSD is essential, both for the health of the parents as well as for the child. Even a few posttraumatic stress symptoms can have a significant influence on various parenting domains, such as the ability to respond in a sensitive manner to the needs of the child or parenting satisfaction, which may all negatively impact the psychosocial functioning of the child [19, 32, 33]. It has indeed been shown in other pediatric populations, such as pediatric cancer, that parental posttraumatic stress symptoms were associated with psychosocial problems (e.g. behavior problems) in the child [34].

One limitation of this study was that the timing of the follow-up measurements was not equal for both parents. One participant (the mother) completed the third measurement at 9 months instead of 3 months post treatment, following several reminders by the researcher. This illustrates that even the completion of questionnaires may take a lot of effort of parents, often overburdened by the intensive and complex care for their ill child. Therefore, the use of brief screening instruments at standard times in future research is recommended.

In conclusion, time-limited EMDR might be an efficient treatment to significantly reduce posttraumatic stress and comorbidity in parents of children with a progressive disease. The small number of parents included in this study does not allow definite conclusions. However, the promising and clinical relevant outcomes of this report should stimulate future studies such as RCTs to validate the efficacy of EMDR for traumatized parents of children with rare progressive disorders.

## Data Availability

Data that support the findings of this study are available from the corresponding author on reasonable request due to privacy restrictions.

## Abbreviations

BSI: Brief symptom inventory
EMDR: Eye movement and desensitization reprocessing
IEM: Inborn error of metabolism
IES-R: Impact of events scale revised
MPS III: Mucopolysaccharidosis type III
PTSD: Posttraumatic stress disorder
RCI: Reliable change index
SUD: Subjective units of disturbance
VOC: Validity of cognition
